# Homoparentalidade e os atendimentos de saúde: narrativas de gays e lésbicas

**DOI:** 10.1590/0102-311XPT030025

**Published:** 2025-12-01

**Authors:** Romeu Gomes, Marcia Tereza Couto

**Affiliations:** 1 Instituto Nacional de Saúde da Mulher, da Criança e do Adolescente Fernandes Figueira, Fundação Oswaldo Cruz, Rio de Janeiro, Brasil.; 2 Universidade de São Paulo, São Paulo, Brasil.

**Keywords:** Homossexualidade, Parentalidade, Pesquisa Qualitativa, Homosexuality, Parenting, Qualitative Research, Homosexualidad, Parentalidad, Investigación Cualitativa

## Abstract

O estudo analisa as relações entre homoparentalidade e os atendimentos de saúde para casais, seus filhos e famílias, considerando serviços de saúde públicos e privados. Por meio dessa análise, pretende-se discutir os sentidos, atribuídos pelas pessoas que vivem em famílias homoafetivas, da busca por saúde e dos atendimentos recebidos. Foi adotada uma abordagem qualitativa, fundamentada na teoria da narrativa e no conceito-chave de *habitus*. Empregou-se entrevistas abertas com uma questão de estímulo para as narrativas de gays e lésbicas. A conjugalidade e a adoção se afiguram como cenários principais. Valorização, desilusão, estranhamento e desqualificação são os principais sentidos associados à conjugalidade. A adoção, por sua vez, se constitui como um dos principais motivos pela busca de atendimento em saúde. Em termos de personagens, destacam-se profissionais de saúde, ora avaliados com positividade, ora com pouco preparo para atender casais de gays ou de lésbicas, envolvidos em situações de estranhamento e desconforto. Os enredos das narrativas, constituídos por eventos e incidentes, figuram como marcos temporais em torno das experiências dos casais na busca por atendimento em saúde. Conclui-se que as relações entre casais homoparentais, e os atendimentos de saúde, nos serviços de saúde públicos e privados podem ser marcadas por desconfortos e estranhamentos.

## Abstract (en)

This study analyzes the relationships between same-sex parenting and healthcare for couples, their children, and families in the context of public and private healthcare services. Through this analysis, we discuss the meanings attributed by individuals living in same-sex families to the search for health care and the care received. A qualitative approach was adopted grounded in narrative theory and the key concept of habitus. Open-ended interviews were used, with a question prompting the narratives of gay and lesbian couples. Conjugality and adoption were key scenarios. Appreciation, disillusionment, discomfort, and disqualification were the main meanings associated with conjugality. Adoption, in turn, was one of the main reasons for seeking healthcare. In terms of social actors, healthcare providers stood out - at times assessed positively, at times seen has having little preparation for providing care to gay and lesbian couples, involving situations of discomfort. The narratives comprised events and incidents, constituting temporal landmarks surrounding the experiences of couples seeking health care. In conclusion, relationships between same-sex couples and health care at public and private health services can be marked by discomfort.

## Abstract (es)

El estudio analiza las relaciones entre homoparentalidad y las atenciones de salud para parejas, sus hijos y familias, considerando servicios de salud públicos y privados. A través de este análisis, se pretende discutir los sentidos atribuidos por las personas que viven en familias homoparentales a la búsqueda de salud y a las atenciones recibidas. Se utiliza un abordaje cualitativo, fundamentado en la teoría de la narrativa y en el concepto clave de *habitus*. Se emplearon entrevistas abiertas con una cuestión de estímulo para las narrativas de gais y lesbianas. La conyugalidad y la adopción se configuran como escenarios principales. Valorización, desilusión, extrañamiento y descalificación son los principales sentidos asociados a la conyugalidad. La adopción, por su parte, se constituye como uno de los principales motivos de la búsqueda de atención en salud. En cuanto a los personajes, destacan los profesionales sanitarios, a veces valorados positivamente, otras veces con poca preparación para atender a parejas gais o lesbianas, involucrados en situaciones de extrañeza e incomodidad. Las tramas narrativas, constituidas por acontecimientos e incidentes, figuran como hitos temporales en torno a las experiencias de las parejas en la búsqueda de atención sanitaria. Se concluye que las relaciones entre las parejas homoparentales y la atención sanitaria, tanto en los servicios públicos como en los privados, pueden estar marcadas por incomodidades y extrañeza.

## Introdução

As configurações familiares contemporâneas, marcadas por uma crescente diversidade, desafiam concepções tradicionais e promovem debates sobre o que, no senso comum, é considerado uma família “normal”. As famílias homoafetivas nos ajudam a refletir - como se fossem óculos que procuramos quando os temos no nariz ^1^ - sobre novas formas de convivência e problematizar a chamada família “normal” no discurso social.

Em 2011, foi sancionada a união de casais homoafetivos ^2^. Essa sanção se deu por meio de um entendimento do Supremo Tribunal Federal sobre o assunto e não por uma mudança na *Constituição Federal* do Brasil. Segundo Barroso ^2^, o artigo 226, no seu inciso 3º, que reconhece a união estável entre homem e mulher, aponta para uma superação da distinção entre casamento e relações de companheirismo, não deve ser interpretado “*como norma excludente e discriminatória, voltada a impedir a aplicação do regime da união estável às relações homoafetivas*” (p. 138). Ainda que haja esse entendimento jurídico, em torno de casais homossexuais surgem dificuldades e, em alguns casos, perplexidades ^3^ ^,^ ^4^.

A esses casais, num cenário social mais favorável, comumente, apenas se concede o reconhecimento parcial de estatuto de união de fato. Esse reconhecimento - ainda que parcial - pode colocar em ameaça a ordem social ou a ordem moral fundada pela norma tácita da união tida como legítima, à qual geralmente se reserva o nome de família ^1^.

No caso de casais homoafetivos que desejam ter filhos por adoção, tecnologia reprodutivas ou pela criação de filhos de um dos parceiros, além da dificuldade social de se atribuir a eles o estatuto de união familiar, se coloca em cena também a discussão do conceito de parentesco, comumente reduzido a laços consanguíneos. Essa associação - consciente ou não -, que se afigura de uma forma mecânica, pode tornar invisível a reivindicação ou o direito desses casais se constituírem como famílias. Para a compreensão dessas famílias, o importante é pensarmos o parentesco para além da consanguinidade, enquanto norma absoluta ou universal que institui o estatuto de família.

Para se pensar para além da consanguinidade, destacamos Sahlins ^5^, que propõe a noção de “mutualidade do ser”, segundo a qual parentes são aqueles que compartilham a vida, vivendo e morrendo uns pelos outros. Assim, na ordem simbólica, o parentesco expressa pertencimento e não uma conexão biológica por “sangue”. Pessoas se tornam parentes de maneiras que vão desde compartilhar o mesmo nome ou a mesma comida até ajudar umas às outras a sobreviver ^5^.

O tensionamento entre ideias conservadoras e novas demandas sobre família e parentesco permite discutir a homoparentalidade. Criado em 1997, pela Associação de Pais Gays e Lésbicas na França, o termo designa qualquer família com pelo menos um adulto homossexual criando um ou mais filhos ^6^. Ribeiro et al. ^7^, com base em Zambrano ^8^, identificam quatro configurações: filhos de relações heterossexuais anteriores; filhos adotivos (formal ou informalmente); filhos biológicos advindos do uso de tecnologias reprodutivas que implicam em coparentalidade, na qual os cuidados com a criança são exercidos de forma conjunta e igualitária pelos parceiros.

Uziel et al. ^9^ observam que o uso do termo homoparentalidade pode reforçar algo que muitas vezes combatemos, que é apontar algo específico na função parental, marcada pela orientação sexual dos pais. Em contraponto, os autores também consideram que o uso desse termo pode politicamente dar visibilidade a essas famílias. Assim, optamos por utilizar o termo para destacar a especificidade da parentalidade envolvendo gays e lésbicas.

As discussões sobre homoparentalidade vêm ganhando espaço na saúde em geral, embora ainda tímida no Brasil ^10^. Gross ^6^ considera que o fato de se conceber o assunto como uma temática recente pode ser rechaçado, uma vez que pais homossexuais existem desde a existência da homossexualidade. A autora observa que há casos em que a atração por pessoas do mesmo sexo só foi descoberta após terem filhos na conjugalidade heterossexual. Já Vecho & Schneider ^11^ apontam que embora dados precisos sejam raros, em 2007, a parentalidade do mesmo sexo nos Estados Unidos envolvia de 2 a 14 milhões de crianças, e em Quebec (Canadá), em 2011, 1.410 crianças viviam com casais do mesmo sexo casados ou em união estável.

Gomes et al. ^10^ destacam que, no âmbito da área da saúde, em geral, predomina a visão heterossexual da parentalidade, desconsiderando a homoparentalidade ou a parentalidade homoafetiva e ainda são necessários estudos socioantropológicos para um maior aprofundamento da temática em questão. Com base nessa perspectiva, este estudo analisa as relações entre homoparentalidade e os atendimentos de saúde para os casais, filhos ou famílias como um todo, prestados por profissionais de saúde da rede pública ou pelo setor privado, a partir de uma perspectiva teórico-conceitual da narrativa. Nesse estudo, levou-se em conta famílias de gays e lésbicas cisgêneros, com identidade de gênero e expressão correspondente ao sexo atribuído no nascimento ^12^.

Neste estudo, as narrativas são tanto objeto quanto método. Para Ricoeur ^13^, a vida sempre pode ser conhecida e expressa pela narração, embora muitas vezes se tente dissociá-la da experiência vivida, confinando-a à ficção. O autor propõe repensar a relação entre história e vida, reconhecendo que a ficção pode ampliar a compreensão da vida humana ^14^. Bruner ^15^ acrescenta que as narrativas são versões da realidade moldadas por convenções culturais e não por verificação empírica ou lógica.

Além da perspectiva teórica conceitual da narrativa, recorremos ao conceito de *habitus* de Bourdieu, entendido como um conhecimento adquirido, um haver, um capital que se relaciona a uma disposição incorporada ^16^ ^,^ ^17^. Por meio desse conceito, é possível articular as dimensões objetiva (estrutura) e subjetiva (percepção, classificação, avaliação); ele não só interioriza o exterior como exterioriza o interior.

O estudo das narrativas de famílias homoparentais e sobre os atendimentos de saúde contribui para compreender as motivações e experiências, podendo suprir lacunas na formação dos profissionais de saúde. Assim, o estudo tem a originalidade de transcender a visão individual de pessoas gays e lésbicas sobre a sua saúde e a relação com profissionais e serviços, oferecendo uma visão abrangente sobre o significado atribuído pelas famílias homoafetivas à busca por saúde e aos atendimentos recebidos, em contextos de serviços públicos e privados.

## Desenho metodológico

Este estudo integra uma pesquisa apoiada pelo Conselho Nacional de Desenvolvimento Científico e Tecnológico (CNPq) sobre homoparentalidade e saúde coletiva, aprovada pelo Comitê de Ética do Instituto Nacional de Saúde da Mulher, da Criança e do Adolescente Fernandes Figueira, Fundação Oswaldo Cruz (protocolo nº 2673/VDP/2022).

O processo desenvolvido ancorou-se numa abordagem qualitativa, buscando investigar os sentidos que os sujeitos atribuem aos fenômenos e ao conjunto de relações em que eles se inserem ^18^. Junto a essa abordagem, adotamos uma posição de que as narrativas podem possuir um método próprio para o seu tratamento analítico. Nesse sentido, utilizamos a proposta de Gomes & Mendonça ^19^, que propõe três instâncias para o estudo das narrativas: (a) compreensão do seu contexto; (b) análise de seus aspectos estruturais (cenários, enredos e personagens); e (c) síntese interpretativa, levando em consideração o diálogo das narrativas com o referencial teórico para interpretá-las.

Para a busca dos narradores, utilizou-se a estratégia de universos familiares, em que pessoas conhecidas do pesquisador, na cidade do Rio de Janeiro, indicam outras a serem entrevistadas e estas, por sua vez, indicam novos entrevistados ^20^ ^,^ ^21^. Na seleção dos narradores, consideramos os seguintes critérios: (a) ser assumidamente gay ou lésbica cisgêneros; (b) ser parte de um casal homoafetivo cisgênero; (c) ter filho(s), participar da criação de filho(s) de parceiros(as) e estar envolvido(a) no processo de tomada de decisão sobre o projeto de parentalidade; (d) ter tido, pelo menos, dois atendimentos em serviços de saúde para si ou para seu(sua) companheiro(a) ou para seu(sua) filho(a).

A produção das narrativas foi facilitada pela utilização de uma entrevista aberta e, para incentivar os entrevistados a desenvolverem seu depoimento, empregou-se a seguinte questão: Quais foram os motivos para buscar atendimento em serviços de saúde para você, para seu(sua) parceiro(a) ou para seu filho(a) e quais foram suas experiências com esse atendimento? Ao final ou durante alguma narração, solicitou-se esclarecimentos de dúvidas sobre algo narrado. A produção da narrativa foi realizada por via remota, com a utilização de recursos da internet. Previamente, os narradores, já contatados por nós por meio do WhatsApp, receberam por e-mail, com o convite formal, o Termo de Consentimento Livre e Esclarecido (TCLE) e ficha com perguntas sobre suas informações pessoais. Todos os narradores nos devolveram o TCLE assinado e a ficha preenchida antes da realização da entrevista.

A nossa intenção era produzir entre 8 e 10 narrativas e, a partir desse quantitativo, analisar se o material era ou não suficiente para o estudo. Após essa análise vimos que seria adequado buscar mais narrativas. Assim, foram contempladas 13 narrativas. A produção das narrativas ocorreu no período de janeiro a abril de 2024. O total de duração do acervo das narrativas foi de 493 minutos, sendo a média de duração de 40 minutos. A maior duração foi de 59 minutos e a menor foi de 22 minutos.

As narrativas produzidas online, no ambiente Google Meet (https://meet.google.com), foram gravadas e, em seguida, transcritas. No tratamento do acervo das narrativas, seguimos as seguintes etapas analíticas: sucessivas leituras do acervo das narrativas; identificação de trechos dos relatos relacionados aos componentes das narrativas (cenários, personagens e enredos); descrição dos achados de cada componente das narrativas e elaboração de síntese interpretativa, estabelecendo articulação entre componentes das narrativas, significados e princípios teórico-conceituais do estudo.

## Resultados do estudo

### Narradores(as) e narrativas

Com base na caracterização dos 13 narradores(as) (Quadro 1), destaca-se o sexo masculino e a cor autorreferida como branca. Quanto à orientação sexual, ao serem convidados para a pesquisa, todos se definiram como homossexuais. Entretanto, Hugo, em sua narrativa, se declarou como bissexual. A média de idade é de 46 anos, sendo a menor idade 34 e a maior 56. Todos os narradores tinham Ensino Superior, sendo que um concluiu mestrado e cinco o doutorado. Quatro são profissionais da área da saúde.

**Quadro 1:** Caracterização dos narradores.

NARRADORES *	SEXO	ORIENTAÇÃO SEXUAL	COR DE PELE REFERIDA **	IDADE (ANOS)	CURSO DE MAIOR GRAU CONCLUÍDO	ÁREA DE ATUAÇÃO	MEMBRO DE ASSOCIAÇÃO OU GRUPO	MUNICÍPIO (ESTADO) DE RESIDÊNCIA
Hermes	Masculino	Gay	Branca	42	Mestrado	Advocacia	Sim	Rio de Janeiro (Rio de Janeiro)
Homero	Masculino	Gay	Branca	43	Especialização	Design	Sim	Curitiba (Paraná)
Helena	Feminino	Lésbica	Branca	48	Residência	Medicina	Não	Rio de Janeiro (Rio de Janeiro)
Hélio	Masculino	Gay	Parda	44	Ensino Superior	Recursos Humanos	Sim	Balsa Nova (Paraná)
Hugo	Masculino	Bissexual	Parda	46	Especialização	Risco Tecnológico	Sim	Curitiba (Paraná)
Hebe	Feminino	Lésbica	Branca	52	Ensino Superior	Fonoaudiologia	Não	Curitiba (Paraná)
Heloisa	Feminino	Lésbica	Branca	34	Ensino Superior	Medicina	Sim	Ribeirão Preto (São Paulo)
Henrique	Masculino	Gay	Preta	38	Doutorado	Magistério Superior	Não	São Carlos (São Paulo)
Heitor	Masculino	Gay	Branca	54	Doutorado	Magistério Superior	Sim	São Carlos (São Paulo)
Heráclito	Masculino	Gay	Parda	49	Ensino Superior	Contabilidade	Não	Niterói (Rio de Janeiro)
Hilda	Feminino	Lésbica	Branca	50	Doutorado	Magistério Superior	Não	São Carlos (São Paulo)
Haroldo	Masculino	Gay	Branca	56	Doutorado	Farmácia	Não	Rio de Janeiro (Rio de Janeiro)
Humberto	Masculino	Gay	Branca	46	Doutorado	Magistério Superior	Não	Manaus (Amazonas)

* Nomes fictícios;

** Classificação adotada pelo Instituto de Brasileira de Geografia e Estatística (IBGE).

Seis narradores pertenciam a uma associação ou a um grupo. Isso se explica pelo fato de o primeiro entrevistado, que indicou outros, ser membro da Associação Brasileira de Famílias Homotransafetivas (ABRAFH). Essa associação, inclusive, publicou um aviso sobre a pesquisa, incentivando seus membros a participarem dela.

O fato de os entrevistados terem sido acessados por meio da estratégia de universos familiares fez com que houvesse algumas características comuns que se configuram em um contexto específico para as narrativas. Pode-se dizer que todas elas trazem um lugar constituído e legitimado, ancorado em posições sociais e capitais simbólicos ^22^. Nas narrativas deste estudo, esse lugar pode ser visto como o de classe média alta.

Na análise das narrativas, não se observou diferenças em termos de acesso aos serviços ou atendimento na área da saúde por cor autorreferida, idade ou grau de escolarização.

### Cenários

Cenários, enredos e personagens se afiguram nas narrativas de uma forma imbricada, a ponto de trazer superposições e indefinições de suas fronteiras.

Os cenários mencionados não são físicos, mas espaços de significação que remetem às “*construções de nichos sociais nas quais relações pessoais e interpessoais específicas são postas a funcionar*” ^23^ (p. 70). Nesses espaços, se constrói simbolicamente as experiências da busca por atendimento de saúde, dois cenários são identificados: parceiros(as) e adoção.

“Parceiros” ou “parceiras”, denominações utilizadas por nós para expressões como casamentos e uniões, ocupam um pequeno espaço no acervo das narrativas. Parceiros(as) são apenas citados sem que sejam desenvolvidos comentários. Em algumas narrativas, a união homoafetiva não é mencionada, a exemplo de Haroldo, que se declarou como “*pai solo*”, por adoção, e não informou ter um parceiro considerado como família na ficha de identificação.

Os sentidos principais que emergem nas narrativas sobre conjugalidade são: a valorização, exemplificada por Hermes, que associa o casamento homoafetivo à libertação de relações de fachada e ao resgate do desejo de paternidade; a desilusão, como relatado por Homero, cuja união de 14 anos terminou em decepção pela infidelidade do ex-parceiro; e o estranhamento, apontado por Hebe, que destaca o impacto social das uniões homoafetivas femininas, hoje mais visíveis e assumidas.

O estranhamento pode causar desconfortos em terceiros. Hélio observa, ao buscar pronto atendimento para seu parceiro, “*as enfermeiras não estavam confortáveis com a situação de eu estar ali com o meu marido*”. O fato de um homem se apresentar como marido de outro homem possivelmente pode causar desconforto, uma vez que casais costumam ser vistos como um par homem-mulher. Nesse sentido, ainda que a conjugalidade seja pouco mencionada pelos entrevistados, ela produz estranhamentos em contextos de atendimento à saúde.

Por último, observa-se o sentido da desqualificação da homoconjugalidade por parte de terceiros. A exemplo disso, Heráclito observa que, apesar de viver com o marido há 27 anos, a sua conjugalidade já foi questionada indiretamente pelo sermão de um pastor evangélico em uma igreja. Ele sente que, pelos comentários de terceiros sobre insinuações de “*cantadas*” na presença de seu parceiro, o casamento dele não é validado pelas pessoas.

Seguindo a linha da desqualificação, Henrique destaca que, mesmo no âmbito profissional, há os que consideram “*a relação homoafetiva como se fosse efêmera* [que iria passar] *rápido, como se não durasse muito tempo*”.

O segundo espaço simbólico que se afigura na maioria das narrativas é a adoção, aparecendo como um dos principais motivos pela busca de atendimentos em saúde. Os sentidos atribuídos a ela são o de valorização. Helena, por exemplo, menciona que “*não se pode adotar por benevolência, por caridade* (...) *é uma escolha de como ser pai ou mãe*”. Já Hermes viu na adoção a “*certeza da paternidade*” e para Haroldo foi uma forma de realizar “*um desejo de ser pai*”.

A adoção, na narrativa de Haroldo, serve como disparador de acontecimentos marcantes. Ele encontrou uma jovem grávida no Nordeste sem nenhuma condição de ter filhos. Os dois decidiram que a criança ficaria sob a guarda dele. Ele trouxe a jovem para o Rio de Janeiro, que realizou seu pré-natal e parto no serviço em que ele trabalha. Depois de passar por um processo de guarda provisória, conseguiu a adoção de sua filha.

Em duas narrativas, a adoção não surge como uma opção para o casal. Henrique e o seu parceiro não pensam em adotar um filho por causa das dificuldades de “*viver uma parentalidade gay na sociedade*”. Já Heitor, que vive há dois anos com o seu parceiro, considera que a decisão de adotar “*deve esperar para que haja maior estabilidade na relação*”.

A busca por atendimento em saúde pode preceder a adoção, como é o caso de Homero, que sentiu necessidade de “*fazer terapia por anos*” para prevenir ou lidar com potenciais questões de saúde mental de crianças adotadas. Homero mencionou que procurou atendimento de psiquiatra para obter um atestado para adotar uma criança. Hélio também precisou de um atestado psiquiátrico, além de um relacionado à saúde em geral, para se candidatar à adoção.

A adoção pode trazer desconfortos quando gays ou lésbicas - ao procurarem serviços de saúde públicos ou privados - são solicitados a darem o nome da mãe biológica da criança. A exemplo disso, Humberto menciona que “*é o único constrangimento que a gente tem até hoje em relação à saúde e que segue com ele hoje por conta de os cadastros obrigarem ter um nome feminino*”.

A narrativa de Hugo sobre a adoção traz uma situação que pode suscitar questionamentos. Segundo ele, quando um gay de seu círculo de amizade buscou adotar uma criança, sugeriram que fosse uma menina. No caso dele e de seu parceiro, também recomendaram uma menina, sem uma justificativa clara. Hugo não traz uma resposta para esse fato. No entanto, essa recomendação levanta a seguinte questão: por que gays devem adotar meninas e não meninos?

### Personagens

No acervo das narrativas, três personagens são mencionados. Dois deles permitem uma caracterização mais detalhada, enquanto o terceiro é citado de forma tangencial. As relações entre narradores e personagens neste contexto marcam momentos que influenciam a experiência de busca por atendimento em saúde.

Nas narrativas sobre adoção, o parceiro é o primeiro personagem destacado nas narrativas, termo que abrange maridos, esposos, esposas, companheiros ou companheiras mencionados pelos narradores. Chama a atenção as menções dos termos “*marido*” e “*esposa*”. Tais menções, ainda que se tratem de uniões homossexuais, podem revelar a internalização da lógica heterossexual como norma.

Geralmente, os parceiros são citados apenas como uma informação, sem aprofundamento nas experiências compartilhadas. Por exemplo, Heráclito, Humberto e Heitor mencionam apenas os nomes de seus maridos, enquanto Hebe descreve sua família como composta por ela e sua esposa. Em algumas narrativas, porém, os parceiros aparecem de forma positiva, como no relato de Hugo, que afirma ter uma relação “*bem tranquila*” com seu marido.

Na narrativa de Helena, a ex-parceira aparece como importante partícipe da relação, uma vez que forneceu seus óvulos para gestar os dois filhos com sêmen de doador anônimo. As duas têm a guarda compartilhada de seus filhos. Heloisa também traz o protagonismo de sua esposa, que abandonou a sua profissão de professora para cuidar de todas as demandas do processo de adoção da filha.

Na narrativa de Homero, o ex-parceiro é mencionado com sentido negativo atribuído a ele. Segundo ele, viveu 10 anos sem saber que o seu ex-marido era “*infiel*”, que mantinha relações paralelas.

O segundo personagem, destacado no conjunto das narrativas, é representado pelos profissionais de saúde, que envolvem - principalmente - médicos, enfermeiras e atendentes. Pelo menos três sentidos são atribuídos a esses profissionais.

O sentido predominante é o de positividade no atendimento. Homero relata que há profissionais de saúde que prestam atendimento de excelente qualidade a ele e aos seus filhos, abordando sem pudor o fato de ele ser gay. Helena destaca a postura acolhedora de um médico que participou da inseminação de sua ex-esposa: para ela, “*ele curtiu o processo*” e chegou a comparecer no aniversário de um ano do filho, gerando comentários bem-humorados de que “*o filho era a cara dele*”. Henrique destaca o bom atendimento de um médico especializado em um serviço como o Centro de Atenção Integral à Criança, descrevendo-o como muito bom. Ele observa que os profissionais de enfermagem e medicina da rede pública são atenciosos e cita um médico recém-formado que “*fez uma anamnese fantástica*”.

Outro sentido atribuído aos profissionais de saúde é o misto de estranhamento e desconforto que ocorre durante o atendimento.

Segundo Hermes, é “*algum grau de estranhamento, mas nada que ultrapasse nas expressões faciais*”. Continua ele: “*às vezes, há momentos em que o profissional de saúde não consegue conciliar as informações que recebe de um cônjuge homoafetivo com o que é solicitado em documentos, a exemplo de fichas de vacinação de crianças, em que é solicitado o nome da mãe*”.

No sentido de desconforto, Hélio menciona que “*as enfermeiras não estavam confortáveis com a situação de eu estar ali com o meu marido* (...) *eu percebi que não estava sendo confortável para a equipe*”.

Humberto menciona que percebeu um desconforto nos médicos quando solicitava a eles um atestado para ficar com o seu filho durante internações. Certa vez, segundo ele, o médico perguntou na presença de seu filho: “*mas por que você vai ficar com ele* (...) *ele não tem mãe?*”. Heráclito também menciona que o médico do plano de saúde ficou meio desconcertado ao saber que ele era gay e disse que ia indicar sua esposa, médica de outra especialidade, mas não indicou.

Por último, o despreparo é associado a alguns médicos e atendentes. Nesse sentido, vários trechos são identificados nas narrativas. Helena menciona que um pediatra “*foi inábil*” porque, mesmo sabendo que seu filho vivia com duas mães, perguntou ao seu filho: “*qual é o time do seu pai?*”. Heloisa também menciona a inabilidade de um médico relatado por uma colega, que foi a uma clínica de fertilização com sua companheira e o médico lhes falou: “*vocês entendem que vocês precisam de um homem para filho?*”. Haroldo, que vive só com a filha, diz que, em geral, os profissionais em questão não estão preparados para atender a filha de um pai gay solo.

O despreparo dos médicos, segundo Henrique, se relaciona mais a “*uma questão geracional*”, uma vez que os profissionais formados há mais tempo “*têm uma dificuldade maior de lidar*” com homoparentalidade, indicando “*a deficiência dos currículos na abordagem das questões que remetem à diversidade sexual, de gênero, étnica, enfim...*”. Por outro lado, ele menciona que, quando vai para o campo de estágio, percebe “*que existe uma dificuldade mesmo de lidar com as questões da parentalidade e das relações homoafetivas*”. Dentro de outra lógica, Hebe considera que por trás do despreparo “*existe um preconceito interno bem exacerbado*” entre profissionais que ela conhece.

Filhos(as) podem ser identificados(das) como o terceiro personagem. Em torno dele, são mencionadas algumas informações pelos narradores. Há casais que têm mais de um filho(a). Em geral, passam a fazer parte da família por meio da adoção. Diferentemente da maioria, Helena tem dois filhos gestados com óvulos de sua ex-mulher, que também foi a gestante, e sêmen de doador anônimo. Elas têm guarda compartilhada.

Em uma narrativa, o filho aparece como uma pessoa com deficiência (PCD), com um quadro de hemiplegia, causada por uma lesão hipóxico-isquêmica no lobo parietal esquerdo. Em outra, a filha tem diagnóstico de TDAH (transtorno de déficit de atenção e hiperatividade) e TOD (transtorno opositor desafiador). Além dos intensos cuidados empreendidos pelos casais para cuidar desses filhos, uma lésbica aponta um grande desafio em cuidar da sua filha negra, mencionada como fruto de “*uma adoção inter-racial*”.

Em algumas narrativas, as lésbicas e os gays informam que não têm filhos e não pretendem se candidatar à adoção. Em uma narrativa, uma lésbica comenta que “*não tem filhos*”, mas tem “*duas pets*”. Em outra, um gay não menciona ter filhos, mas convive muito bem com as filhas do casamento hetero anterior de seu marido.

Apesar de as informações acerca das proles serem escassas, em algumas narrativas, elas figuram como protagonistas centrais. Em algumas dessas, esse protagonismo se explica pelo sentido que confere ao fato de lésbicas e gays se constituírem como uma família. É um personagem que é mencionado como alguém que recebe muito afeto. Como menciona Hermes, “*são filhos pelas vias do afeto*”.

### Enredos

Os enredos que emergem nas narrativas aqui analisadas se caracterizam como uma síntese de elementos heterogêneos ^14^, que se configuram por meio de eventos e incidentes. Nesse sentido, as buscas por atendimentos em serviços de saúde em geral ou por atendimentos específicos por parte de profissionais de saúde figuram como marcos temporais em torno dos quais as experiências são narradas.

Os motivos que levam os narradores(as) a buscarem atendimento em saúde para si, para seus parceiros ou para seus(suas) filhos(as) se referem tanto à prevenção, quanto à intervenção. Essa busca ocorre em serviços públicos e privados. O setor público nem sempre é utilizado por alguns porque há dificuldades na conciliação de horários, fazendo com que seja difícil conjugar a rotina de trabalho deles com a disponibilidade de horários nesses serviços. A narrativa de Hermes ilustra bem as dificuldades que podem surgir no acesso a serviços públicos, principalmente nos setores secundários ou terciários. Segundo ele, no período inicial de adoção de seu filho mais velho, teve recorrentes dificuldades no acesso ao atendimento desse filho. A juíza determinou que os atendimentos em saúde fossem realizados num hospital de referência longe de sua casa. O longo trajeto de sua casa para esse hospital era realizado por ônibus. Quase sempre, ele tinha que ir em pé com o filho no colo e a bolsa de bebê. Ninguém cedia um assento para ele.

Em algumas narrativas, pode-se apreender que os serviços públicos devem ficar para quem mais precisa. Concorrendo com essa ideia, Heitor destaca que há necessidade de se “*desconstruir aquela visão de que o SUS* [Sistema Único de Saúde] *é só para pessoas de baixa renda*”. Observa que, em muitas áreas - como na assistência a pessoas com HIV - o SUS é melhor do que a rede privada. Ele e seu companheiro conseguem atendimento e são bem recebidos.

Não se observa, no acervo das narrativas, uma clivagem entre as experiências em serviços públicos e em serviços privados, em termos de positividade e negatividade. Em ambos os tipos de serviços destacam-se tanto experiências positivas quanto negativas.

Em geral, nos enredos, os incidentes assumem o primeiro plano. Assim, na composição da narração, destacam-se aqueles que, de certa forma, apresentam estranhamentos gerados por situações que se polarizam: de um lado, necessidades e cuidados homoparentais que impulsionam a busca por atendimentos em saúde e, de outro, modelos heterossexuais comumente incorporados como referência de família que se projetam na relação entre quem busca atendimento e quem presta atendimento.

Relacionado a esse estranhamento, um episódio narrado por Hermes pode ser ilustrativo. Ele destaca que houve “*algum grau de estranhamento de uma ou outra pessoa* [que prestou o atendimento]*, mas nada que ultrapassasse o estranhamento nas expressões faciais*”. Mencionou que, ao entregar a ficha de cadastro de vacinação de seu filho mais velho, foi questionado por uma atendente sobre o fato de não constar o nome da mãe. Frente a esse estranhamento, ele respondeu para a atendente que o seu filho não tinha mãe, mas tinha dois pais. Como reação, segundo ele, a atendente “*fez um bico!*”. Em seguida, ele arremata: “*eu devo ter ofendido alguns valores religiosos, morais, de credo, sei lá. Afetei ela de alguma forma*”.

Os estranhamentos por parte de quem presta atendimento, às vezes, causam desconfortos em quem busca o atendimento. Sobre isso, Hermes menciona que sente desconforto quando perguntam “*onde está a mãe?*”. Segundo ele, ao dizer que ia ficar junto de seu filho, um médico - na frente da criança - indagou “*mas ele não tem mãe?*”. Como resposta, ele “*tinha que explicar que era um caso de paternidade solo e que* [era ele quem] *cuidava dele. Aí, as pessoas me olhavam com uma cara estranha*”.

O estranhamento em lidar com a homossexualidade pode fazer com que o profissional de medicina se iniba a ponto de não conduzir a consulta conforme um padrão esperado. Heráclito, por exemplo, conta que um médico de plano de saúde ficou “*desconcertado*” ao saber que ele era gay. Hilda menciona que, ao descobrir que a sua esposa era lésbica, uma médica “*paralisou* (...) *não soube conduzir a consulta, enfim, houve um certo mal-estar* (...) *e ela não teve como conduzir adiante o tratamento, os exames que deveriam ser feitos etc.*”.

Heráclito conta que, ao falar para o médico que era gay e pretendia fazer uso da PrEP (profilaxia pré-exposição), foi desaconselhado de forma rápida, incisiva e sem explicação a não fazer isso. Segundo ele, a recepcionista ouviu e “*ficou apavorada com o atendimento que ele* [médico] *deu. Depois, em particular, ela falou para mim que eu deveria fazer uso sim da PrEP como medida preventiva*”.

Heloisa observa que a sua profissão pode minimizar as reações de outros médicos. Segundo ela, as pessoas “*que já sabem que eu sou médica, então já se sentem mais acuadas, já ficam mais na defensiva e já se policiam para não pecar*”. Ao descobrir que são duas mulheres que convivem, às vezes aceitam essa relação. Mas isso não a impede de viver situações que possam trazer constrangimentos: “*as partes mais chatas é a gente se apresentar, passar pela triagem, passar pela secretária, passar pelo preenchimento de uma ficha, esse tipo de conduta*”.

Junto aos incidentes, há também acontecimentos considerados positivos por parte de quem busca atendimentos. Homero, por exemplo, menciona acontecimentos positivos relacionados à vacinação de seus filhos ou à obtenção de medicação gratuita nos espaços públicos de atendimento de saúde, destacando o SUS como sendo importante para a saúde em geral. Henrique observa que nunca sentiu “*nenhuma situação de preconceito*”. Um acontecimento de destaque para ele foi o de ser atendido em um Centro de Atendimento a Doenças Infectocontagiosas (CAIC). Para surpresa dele, o seu atendimento foi com “*delicadeza e atenção*”. Sua experiência recente ocorreu nesta grata surpresa, com esse serviço público de saúde. Hélio também menciona ter recebido, junto com seu marido, um atendimento atencioso no setor privado de saúde.

### Síntese interpretativa

Os sentidos das narrativas de gays e lésbicas analisadas neste estudo emergem no encontro entre o que é contado pelos narradores e a escuta e interpretação de suas histórias. Ampliando a perspectiva de Ricoeur ^14^, consideramos que essas narrativas se tornam inteligíveis não apenas de forma isolada, mas como parte de um conjunto analisado coletivamente.

Partindo dos pressupostos de Schutz ^24^, os narradores aqui reunidos tipificam um grupo de casais homossexuais, de classe média alta e de uma capital brasileira que, em grande parte, busca constituir famílias, muitas vezes, incluindo filhos por meio da adoção ou de tecnologias reprodutivas. A interconexão desses narradores, acessados por indicação mútua, conforme evidenciado na metodologia utilizada, evidencia sua inserção em um universo material e simbolicamente compartilhado.

As experiências comuns vivenciadas por esses sujeitos na busca por atendimentos de saúde, seja no setor público ou privado, permitem a construção de uma metanarrativa que transcende as narrativas individuais. Essa metanarrativa funciona como uma trama, composta por fios ou enredos que refletem tanto permanências quanto rupturas em relação às normas sociais de gênero e parentalidade.

Nesse contexto, a análise à luz do conceito de *habitus* de Bourdieu ^17^ ^,^ ^18^ ilumina como essas normas de gênero são internalizadas ou contestadas pelos narradores em suas vivências. Alternativamente, pudemos explorar como as dinâmicas sociais e culturais moldam a percepção e o tratamento recebidos pelas famílias homoparentais nos serviços de saúde.

Esse tratamento, tanto pela compreensão dos enredos aqui tratados, quanto pela forma como os personagens profissionais de saúde são representados, pode ser associado à invisibilidade e/ou exclusão dessas famílias nos espaços dos atendimentos de saúde. Isso pode ser explicado pelo modelo que Bourdieu denomina de “*ordem heteronormativa*” ^25^ (p. 146). Esse modelo - que estrutura a forma como se vê e pensa a sexualidade como única e exclusivamente de ordem heterossexual - pode fazer com que haja um despreparo dos médicos ao lidarem com parceiros ou parceiras do mesmo sexo. Se já é difícil lidar com as pessoas que contestam a hegemonia heteronormativa, lidar com pares que contestam o modelo de família heterossexual amplamente naturalizado parece ser um desafio ainda maior.

Por outro lado, no caminho de Bourdieu ^1^ ^,^ ^17^ ^,^ ^25^, compreendemos que os aspectos culturais e políticos vinculados à posição social de classe e presentes nos repertórios dos narradores podem influenciar a sua busca por saúde, a partir da noção de “famílias legítimas”, desafiando intencionalmente o discurso social. Nesse sentido, ainda que considerem a existência de barreiras simbólicas e práticas no cotidiano dos serviços e no atendimento ofertado pelos profissionais de saúde, eles se afirmam como famílias legítimas. Assim, por ser dinâmico, o *habitus* ao mesmo tempo em que se revela como permanência, também é fonte de ações que buscam mudanças.

Bourdieu ^17^ observa que uma das funções do *habitus* é descartar duas teorias: uma que entende a ação como um efeito mecânico da coerção de causas externas e a outra que defende a ideia de que os agentes das ações atuam de forma livre e consciente, entendendo a ação como o produto de cálculo das chances e dos ganhos.

A construção da parentalidade e da conjugalidade homoafetiva em um contexto marcado pela hegemonia heteronormativa, tal como se apresenta na sociedade brasileira, mesmo em um centro urbano como o Rio de Janeiro, se configura, assim, como um campo de contendas simbólicas, no qual os sujeitos buscam ressignificar e legitimar suas experiências no interior dos serviços de saúde. Nesse processo, observa-se que, embora alguns profissionais expressem resistência ou despreparo, há também aqueles que oferecem acolhimento e atendimento qualificado, indicando que a dinâmica social não se dá de forma homogênea, mas sim em meio a tensões e contradições que refletem transformações mais amplas na sociedade.

Por fim, ao mobilizarem diferentes formas de capital - social, cultural e simbólico - os casais homoparentais participantes deste estudo demonstram que suas experiências não se restringem à mera adaptação às normas vigentes, mas incluem também esforços ativos de contestação e mudança. O acesso à saúde, portanto, emerge não apenas como um direito fundamental, mas também como um espaço de afirmação e reconhecimento de novas configurações familiares.

## Conclusão

As relações entre casais homoparentais e os atendimentos de saúde, nos serviços de saúde públicos e privados, podem ser marcadas por desconfortos e estranhamentos. Isso, em parte, pode ser tributado à hegemonia do modelo heterossexual que influencia tanto a concepção de parentalidade, quanto as interações afetivo-sexuais em geral. Práticas e experiências que escapam dessa normatividade podem ser invisibilizadas ou até mesmo desqualificadas no espaço dos serviços de saúde. No entanto, essa realidade não impede que tais vivências se tornem mais visíveis e discutidas, impulsionando mudanças que promovam maior inclusão e sensibilidade às diversidades de gênero e sexualidade.

Os enredos construídos a partir das narrativas analisadas evidenciam a coexistência de acolhimento e resistência no atendimento a gays e lésbicas. Enquanto alguns profissionais demonstram preparo e sensibilidade no cuidado às famílias homoparentais, outros revelam desconhecimento, desconforto ou até preconceito, dificultando o acesso pleno e humanizado à saúde. Assim, a tensão entre *habitus* e mudança se manifesta de forma concreta nas interações entre os casais homoparentais e o sistema de saúde, reiterando a importância de políticas e práticas que contemplem a pluralidade das formações familiares. A inclusão de conteúdos sobre diversidade sexual e de gênero na formação médica e na capacitação das equipes de saúde pode contribuir para a redução de barreiras simbólicas e estruturais, promovendo um atendimento mais equitativo e qualificado.

## Data Availability

Os dados de pesquisa não estão disponíveis.
